# Association of Household Food Insecurity with Nutritional Status and Mental Health of Pregnant Women in Rural Bangladesh

**DOI:** 10.3390/nu13124303

**Published:** 2021-11-28

**Authors:** S. M. Tafsir Hasan, Daluwar Hossain, Faysal Ahmed, Md Alfazal Khan, Ferdousi Begum, Tahmeed Ahmed

**Affiliations:** 1 Nutrition and Clinical Services Division, icddr,b, Dhaka 1212, Bangladesh; daluwar.hossain@icddrb.org (D.H.); faysal.ahmed@icddrb.org (F.A.); fazal@icddrb.org (M.A.K.); tahmeed@icddrb.org (T.A.); 2 Health System and Population Studies Division, icddr,b, Dhaka 1212, Bangladesh; 3 Department of Gynaecology and Obstetrics, Ibrahim Medical College, Dhaka 1000, Bangladesh; fbegum9@gmail.com; 4 Office of the Executive Director, icddr,b, Dhaka 1212, Bangladesh

**Keywords:** food security, height, weight, MUAC, depression, anxiety, stress, propensity score matching

## Abstract

Food insecurity may affect women’s health; however, pertinent research is scant among pregnant women. This study investigated the association of household food insecurity (HFI) with the nutritional status and mental health of 672 early-gestation (5–16 weeks) pregnant women with a singleton fetus, who participated in the screening activity of a community-based trial (NCT04868669) in Matlab, Bangladesh. Height (cm), weight (kg), body mass index (kg/m^2^), mid-upper arm circumference (MUAC) (cm), depression, anxiety, and stress were the outcomes studied. HFI was assessed using the Household Food Insecurity Access Scale. Women’s depression, anxiety, and stress were assessed using the Depression, Anxiety, and Stress Scales-21. Propensity score matching based weighted multivariable linear and logistic regression were used to evaluate the independent association of HFI with the outcomes. In adjusted models, pregnant women from food-insecure households in rural Matlab were on average 2.0 cm shorter (β = −2.0, 95% CI: −3.3, −0.7), 2.0 kg lighter (β = −2.0, 95% CI: −3.4, −0.7), and had 0.6 cm lower MUAC (β = −0.6, 95% CI: −1.1, −0.1) than their food-secure counterparts. HFI was associated with higher odds of depression (OR = 3.3, 95% CI: 1.8, 5.9), anxiety (OR = 6.1, 95% CI: 3.7, 10.0), and stress (OR = 4.8, 95% CI: 1.6, 14.2) among the women. Public health measures should focus on ensuring proper nutrition during the critical growth periods of life, pregnancy, and external environmental shocks, to mitigate the adverse effects of HFI on women’s health.

## 1. Introduction

At the household level, “food insecurity exists whenever the availability of nutritionally adequate and safe foods or the ability to acquire acceptable foods in socially acceptable ways is limited or uncertain [1].” According to the latest estimate by the Food and Agriculture Organization (FAO), one in ten people in the world experiences severe food insecurity, corresponding to nearly 750 million individuals [2]. In Bangladesh, one in four people remains food-insecure [3]. In rural spheres of low- and middle-income countries (LMICs), including Bangladesh, women are more susceptible to the adverse consequence of household food insecurity (HFI) as they have the smallest share of the meal [2,4]. Women of reproductive age, especially in pregnancy and lactation, are of particular concern because nutritional demands increase in these periods [5], and food insecurity in this population group may also affect the offspring’s growth and development [6,7,8,9,10]. Furthermore, the COVID-19 pandemic has placed reproductive-aged women at remarkably magnified risk of acute food insecurity and its health-related sequelae, including psychological distress and acute undernutrition [11,12,13,14].

Recent studies linked HFI to malnutrition among women in low to high-income countries; however, data are mixed regarding the direction of malnutrition, and the associations are equivocal at best [15,16,17,18,19,20]. HFI was associated with underweight status [15,21,22] and low mid-upper arm circumference (MUAC) [21] in non-pregnant, reproductive-aged women in Ethiopia. Young et al. showed that HFI was associated with lower body mass index (BMI) at 12–28 weeks of gestation among HIV-infected pregnant women in Uganda [23]. Conversely, HFI was associated with obesity among Lebanese mothers [24]. In Mexico, the odds of concurrent anemia and being overweight were higher among reproductive-aged adult women from food-insecure households [18]. Hernandez et al. showed that, in the US, food insecurity was associated with being overweight or obese in white and Hispanic adult women, but not in black women [17]. Another recent study showed that food insecurity was associated with being overweight or obese in Mexican American women, but not in non-Mexican Hispanic women in the US [19]. More interestingly, Castañeda et al. found no association of food insecurity with BMI in female farmworkers in Mexico [16]. Some studies demonstrated an association between HFI and women’s short stature [20,25,26,27], probably a consequence of chronic food insecurity. Additionally, food insecurity may affect women’s mental health and subjective well-being. Studies showed an association between HFI and stress [28], distress [29], depression [30,31,32,33], and poor quality of life [34] in peripartum women, and stress [35,36], anxiety [36], depression [32,35], symptoms of posttraumatic stress disorder [36], and ethnospecific illnesses [35] in non-peripartum women.

Studies on the relationship between HFI and women’s health are scarce in Bangladesh as well as in other LMICs despite the higher prevalence of food insecurity at all levels among women than men [2,13]. Apart from a few mental health studies [28,29,32,34], investigating the effect of HFI in pregnant women, one of the most vulnerable population groups, are also limited, whereas millions of women are exposed to food insecurity in pregnancy [2,37]. Furthermore, few contemporary food-security studies investigated pregnant women in early pregnancy, whereas women’s nutritional status (BMI and MUAC) in the first trimester determines the required gestational weight gain (GWG) and subsequently affects perinatal outcomes [38,39,40,41]. This study investigated the association of HFI with nutritional status and mental health of early-gestation pregnant women in rural Bangladesh.

## 2. Materials and Methods

### 2.1. Study Setting, Population, and Data Source

Data were extracted from the screening dataset of a community-based trial entitled ‘Improving Maternal Nutrition in Matlab (IMNiM)’ (NCT04868669). In the trial, a total of 683 newly pregnant (ultrasound confirmed) women from selected areas of Matlab, a rural subdistrict of Bangladesh, were consecutively screened in their early gestation (5–16 weeks) between January 2020 and January 2021.

The study setting has been described elsewhere [42,43]. In brief, Matlab is located 55 km southeast of the capital, Dhaka. Agriculture, forestry, fishery, and trade are the major occupations of men in Matlab. Most women are housewives. In Matlab, icddr,b (formerly the International Centre for Diarrhoeal Disease Research, Bangladesh [ICDDR,B]) runs a Health and Demographic Surveillance System (HDSS) covering a population of 230,000 [42]. Data on vital events such as births, deaths, marriages, and migrations are updated by community health research workers (CHRWs) through regular home visits. The surveillance area is divided into a government service area and an icddr,b service area where the IMNiM study was conducted. The icddr,b service area is further divided into four blocks. In these areas (blocks), icddr,b offers free-of-cost health services, including maternity and child healthcare, through a large central health facility (Matlab Hospital) and a subcenter in each block. Although there are government-run primary healthcare facilities, women from this area preferentially seek antenatal care (ANC) and maternity services from the icddr,b facilities. Women visit Matlab Hospital for ANC in the first trimester, especially for pregnancy ultrasound and routine lab tests.

The screening started in January 2020 but abruptly stopped when the Government of Bangladesh imposed a two-month-long countrywide lockdown (restriction on movement) from 26 March 2020 to curb the spread of COVID-19 infections in the community [11]. With adequate precautions and special permission of the local administration, we resumed the screening activity on a limited scale in the last week of April 2020. The activity regained pace in May and continued up until January 2021. Before the lockdown, CHRWs identified new pregnancies based on the history of amenorrhea and positive urine strip test during monthly home visits. When we restarted the screening activity after the pause, CHRWs had to depend on women’s over-the-phone self-reporting of amenorrhea as the monthly home visits were discontinued amid the COVID-19 pandemic. The screening took place at Matlab Hospital when the reported pregnant women visited the facility in the first trimester for ANC. The screening procedures involved taking anthropometric measurements, assessing household food insecurity and mental health, and obtaining information on the presence of chronic diseases, pregnancy status, gestational age, dietary habit (vegetarianism), and sociodemographic variables using a pretested semi-structured questionnaire. Women were approached for screening if they were found pregnant on ultrasound. Obstetric and clinical information was recorded after consultation with duty physicians at Matlab Hospital. Gestational age was calculated based on the reported date of the last menstrual period (LMP) and confirmed by pregnancy ultrasound. The accurate age of the women was retrieved from the HDSS database.

From the screening dataset, women were excluded if they were carrying multiple fetuses (*n* = 7), practiced vegetarianism (*n* = 2), or belonged to a household from which a woman was screened earlier (*n* = 2). Thus, for the final analysis, we considered 672 pregnant women.

### 2.2. Household Food Insecurity

Household food insecurity was assessed using the Household Food Insecurity Access Scale (HFIAS), developed by USAID’s Food and Nutrition Technical Assistance (FANTA) III Project [44]. The scale has been used with success in low- and middle-income countries (LMICs), including Bangladesh [11,45], to differentiate between food secure and insecure households. The scale focuses on the access component of food insecurity at the household level. The HFIAS categorizes households into four levels of household food insecurity: food secure; and mild, moderately, and severely food insecure. In this study, the mild, moderate, and severe food insecurity categories were merged into one ‘food insecure’ group due to the relatively small numbers of women in each of the three categories. The tool calculates household food insecurity by asking nine questions, each having four response options in a recall period of 30 days. Higher scores indicate a higher degree of household food insecurity. The details of the tool can be found in the “Household Food Insecurity Access Scale (HFIAS) for Measurement of Food Access: Indicator Guide, version 3” [44]. The HFIAS questions were translated into Bengali and reverse translated, piloted in 26 women, and optimized to ensure all questions were understood by the pregnant women and were culturally appropriate. Trained interviewers administered the tool, and all questions were directed to the pregnant women.

### 2.3. Mental Health

Symptoms of depression, anxiety, and stress in pregnant women over the past week were assessed using the Depression, Anxiety, and Stress Scales-21 (DASS-21) [46]. The DASS-21 is a validated, widely used, standardized self-report scale that can measure three related negative states of mental health such as depression, anxiety, and stress in clinical and non-clinical populations [47,48]. The DASS-21 scale has seven items in each subscale category of Depression, Anxiety, and Stress symptoms. Responses are scored on a 4-point Likert scale ranging from 0 (did not apply to me at all) to 3 (applied to me very much/most of the time), with higher scores indicating higher levels of depression, anxiety, or stress. The DASS depression and anxiety subscales are convergent with the Beck Depression Inventory (r = 0.74) and the Beck Anxiety Inventory (r = 0.81) [49]. The subscales have demonstrated excellent internal consistency, with Cronbach’s alpha of 0.81 for depression, 0.73 for anxiety, and 0.81 for stress [46]. Each subscale of the DASS-21 has different cut-offs to suggest the presence and severity of depression, anxiety, and stress. The details of the tool can be found on the DASS website (www.psy.unsw.edu.au/dass, accessed on 1 October 2021) and in the DASS Manual [46]. The validated Bengali version of the DASS-21 [50] was used in this study. The tool was piloted in 22 women and further optimized to ensure all statements were understood by the pregnant women and were culturally appropriate. The DASS-21 is a self-report instrument, and no special skill or training is required to administer it. Women read and recorded the response themselves; however, in a few women who could not read Bengali, a trained interviewer read out the statements to the women and recorded their response.

### 2.4. Anthropometry

Anthropometric assessment for pregnant women was conducted by trained health workers, using standard techniques and equipment. Women were weighed on a calibrated digital scale (Tanita HD-661, Tokyo, Japan) to the nearest 0.1 kg wearing light clothing and without shoes. The scale was calibrated every day using standard weights. Standing height was measured to the nearest 0.1 cm, without shoes or headwear, using a portable stadiometer (Seca 213, Hamburg, Germany). MUAC was measured to the nearest 0.1 cm using an adult MUAC measuring tape (non-stretchable plastic) on the left arm while the arm relaxed along the body trunk.

### 2.5. Outcomes

This study examined several aspects of the nutritional status and mental health of early-gestation women. Nutritional status was operationalized as height (cm), weight (kg), BMI (kg/m^2^), and MUAC (cm). BMI was calculated as weight (kg) divided by the square of height (m^2^). Height reflects earlier life experience [26], while weight, BMI, and MUAC indicate current nutritional status [51,52]. We also assessed three related mental health outcomes, namely depression, anxiety, and stress. Depression, anxiety, and stress are indicators of psychological distress and negative states of mental health [14].

### 2.6. Covariates

Covariates included sociodemographic and clinical characteristics, such as age, religion, education, parity, gestational age, chronic disease, area (block), and period. Age (years), education (completed years of schooling), and gestational age (weeks) were continuous variables. We recoded parity into three categories: nulliparous, 1 previous birth, or ≥2 previous births. Chronic disease was defined as the presence of hypertension, diabetes mellitus, thyroid disease, rheumatoid arthritis, systemic lupus erythematosus, inflammatory bowel disease, rheumatic fever, or bronchial asthma. The study period was divided into four meaningful sub-periods, namely January–March 2020 (early COVID-19 days), April–June 2020 (first lockdown period), July–September 2020 (post-lockdown period), and October 2020–January 2021 (low infection-rate period). The selection of covariates (and their categorization) was guided by previous studies on risk factors of the study outcomes and studies investigating their associations with food insecurity [17,18,20,26,29,33,35,53].

### 2.7. Statistical Analysis

We began our analysis with descriptive statistics of all women in our dataset. We presented the characteristics of the women in the sample as mean (standard deviation, SD) for continuous variables and frequency measures for categorical variables. As described above, HFI was considered a binary measure. Using an independent two-sample *t*-test, we compared the proportion of individual categories (for categorical variables) and the mean (for continuous variables) of each characteristic (covariate) between the food-secure and food-insecure women. The two-sample *t*-test was used to compare the proportions, before and after matching (described later), based on the recommendations of D’Agostino et al. [54], Upton [55], and Schafer and Kang [56]. As anticipated, in the full (unmatched) sample, the two groups of women tended to differ for most characteristics. Moreover, due to several reasons (described later), the chances of women being screened during the study period were possibly higher for those from food-secure households than those from food-insecure households. The imbalance between the food-insecure and food-secure groups in terms of sample sizes and covariate patterns and the issue of potential selection bias in the full sample must be addressed to assess the unbiased and independent association of HFI with the study outcomes. Given the nature of the dataset, traditional adjustment for covariates in regression models alone is unlikely to yield unbiased estimates [57]. Hence, to address these imbalances and potential confounding in estimating the association between HFI and the study outcomes, we used a doubly robust two-step propensity score matching-based approach [58,59,60]. 

In step 1, we first estimated propensity scores (likelihood of being food-insecure) of women from a logistic regression model in which food insecurity was the outcome of interest and covariates were such that they potentially influence both food security status and the study outcomes (Table S1). We then used radius matching to create matched groups of food-insecure (treated) and food-secure (control) women. The matching was performed without replacement on the estimate of the logit of the propensity scores within a caliper equal to 0.2 times the standard deviation of the logit of the propensity scores [57,61]. Radius matching approach was adopted because it used all observations within the caliper for matching and resulted in an analytic sample comprised of women in the food-insecure and food-secure groups whose propensity scores (covariate patterns) were statistically comparable. We visually checked the overlap of the density distribution of the estimated propensity scores among food-insecure and food-secure women before and after matching (Figure S1). A substantial overlap of the density distribution of the propensity scores between the two groups was observed after matching. Further analysis was restricted to the region of common support, where the propensity scores of the two groups overlapped (Figure S2). The standardized percentage bias, the percentage difference of the sample means in the two groups as a percentage of the square root of the average of the sample variances, was used to check the balance of individual covariates between the two groups before and after matching (Figure S3) [62]. In the matched sample, all the covariates had a standardized percentage bias of less than or around 5%, indicating a satisfactory balance between the two groups. The balancing in the matched sample was also examined using two-sample *t*-tests by comparing the mean or proportion of the covariates (Table 1). Finally, analytical weights were generated for each observation based on the matching status. Propensity score matching was carried out and the weights were generated using the psmatch2 package in Stata [63]. 

Step 2 included estimating the association of HFI with the study outcomes using weighted regression models, using the weights generated in step 1, among the women in the matched sample. This approach was based on the recommendations of Stuart [61], who suggests that, after propensity score matching, it is preferable to “pool all the matches into matched treated and control groups and run analyses using the groups as a whole, rather than using the individual matched pairs [58].” Coupling matching with a regression model is likely to improve the precision of estimates by accounting for any residual confounding not addressed through the matching process. The abovementioned technique has been used previously in community-based studies [60]. In the second step, weighted simple and multiple linear regression models were fitted to assess the association of HFI with height, weight, BMI, and MUAC. We expressed the strength of association as mean difference (β) with a 95% confidence interval (95% CI). To assess the association of HFI with depression, anxiety, and stress, weighted simple and multivariable logistic regression models were fitted. We expressed the strength of association as odds ratio (OR) with 95% CI considering women from food secure households as the reference group. All the multivariable models were adjusted for the covariates of a priori interest, such as age, religion, education, parity, gestational age, chronic disease, area (block), and period.

All statistical tests were two-sided, and statistical significance was evaluated at *p* < 0.05. Data analysis was performed in Stata v15.1 (StataCorp, College Station, TX 77845, USA).

## 3. Results

The full (unmatched) sample included 672 pregnant women before matching, of whom the majority (84.4%) were Muslim, 34.2% were nulliparous, and 4.9% had chronic diseases. The mean (SD) age, education, and gestational age of the pregnant women were 25.3 (5.8) years, 8.2 (3.0) years, and 9.8 (1.8) weeks at the time of screening, respectively. The mean (SD) height, weight, BMI, and MUAC were 152.5 (5.4) cm, 52.6 (9.4) kg, 22.6 (3.8) kg/m^2^, and 26.8 (3.4) cm, respectively. In the full sample, 7.0%, 11.9%, and 2.5% of women reported symptoms suggestive of depression, anxiety, and stress, respectively. Of the 672 women, 71 (10.6%) were from food-insecure households, and 601 (89.4%) were from food-secure households. In the full sample, the prevalence of mild, moderate, and severe food insecurity was 6.1%, 3.4%, and 1.0%, respectively. In the matched sample, 665 pregnant women were retained; 70 food-insecure women were matched to 595 food-secure women.

We observed several differences between the food-insecure and food-secure groups before matching. Compared to the food-secure women, their food-insecure counterparts were slightly older, had lower educational attainment, were screened earlier in pregnancy, were more likely to be nulliparous and from block B, and less likely to be from block C and screened during the period of October 2020 to January 2021. After the groups were matched and matching weights were applied, the abovementioned differences were no longer observed, and the women from the two groups were statistically comparable in terms of characteristics (Table 1).

**Figure 1:**
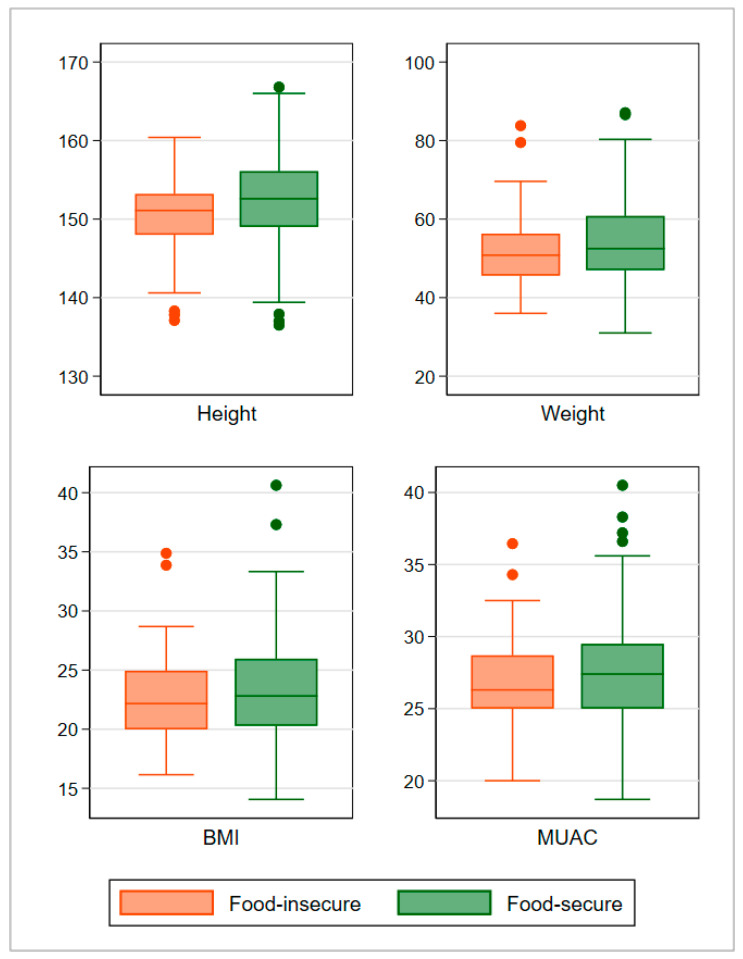
Height (cm), weight (kg), BMI (kg/m^2^), and MUAC (cm) among early-gestation pregnant women in Matlab, after matching (weighted), by household food security status.

Figure 1 visualizes the distribution of height, weight, BMI, and MUAC by food-security status in the matched sample. The average height, weight, BMI, and MUAC tends to be lower among the women in the food-insecure group. 

**Figure 2:**
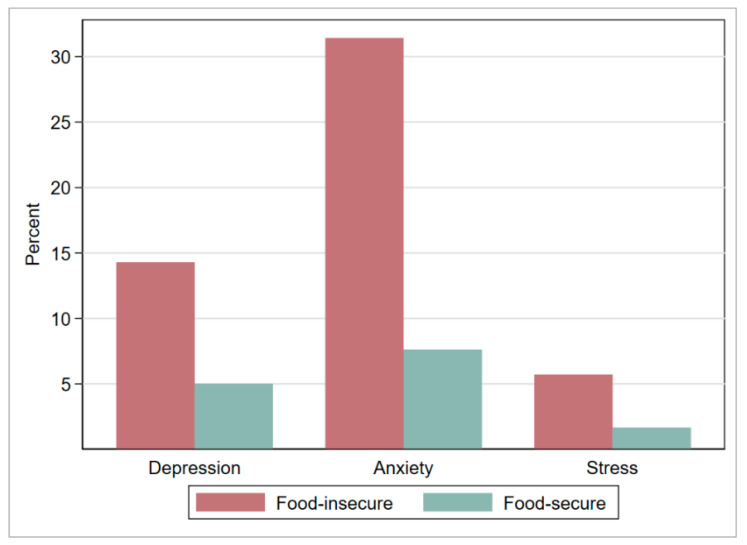
Proportion (percentage) of depression, anxiety, and stress among early-gestation pregnant women in Matlab, after matching (weighted), by household food security status.

Figure 2 shows that the proportion (percentage) of depression, anxiety, and stress was much higher among the food-insecure women compared to their food-secure counterparts. 

Table 2 shows the association of HFI with aspects of women’s nutritional status. In the adjusted models, pregnant women from food-insecure households were on average 2.0 cm shorter (β = −2.0, 95% CI: −3.3, −0.7), 2.0 kg lighter (β = −2.0, 95% CI: −3.4, −0.7), and had 0.6 cm lower MUAC (β = −0.6, 95% CI: −1.1, −0.1) than their food-secure counterparts. Although the association between HFI and BMI shows a negative trend (β = −0.3, 95% CI: −0.8, 0.2) like other nutritional measures, the association was not statistically significant.

Table 3 shows the association of HFI with women’s mental health. In the adjusted models, HFI was associated with higher odds of depression (OR = 3.3, 95% CI: 1.8, 5.9), anxiety (OR = 6.1, 95% CI: 3.7, 10.0), and stress (OR = 4.8, 95% CI: 1.6, 14.2).

## 4. Discussion

The present study is the first to investigate the association of HFI with several aspects of nutritional status and mental health of early-gestation women in rural Bangladesh. We observed that the food-insecure women in Matlab were, on average, 2.0 cm shorter than their food-secure counterparts. Tayie et al. had a similar observation in a nationally representative sample of American women aged 18–50 years; compared to women from fully food-secure households, those from homes with marginal food security were 1.3 cm shorter [26]. Similarly, in a nationally representative sample of Ecuadorian women of reproductive age, household food insufficiency was associated with a 1.7 times higher prevalence of short stature (height < 145 cm) [27]. Our results suggest, although cannot prove due to the cross-sectional nature of the study, that long-term food insecurity, intake of protein-deficient food, and poor dietary diversity, including during the critical growth periods, might have led to stunted linear growth and prevented from reaching the full potential of adult height [26,27,64].

Short maternal height has serious public health implications. Maternal short stature increases the risk of poor GWG [40], obstructed labor [65], caesarean delivery [66], preterm birth, and low birth weight (LBW) [67]. Moreover, short stature is associated with reduced work capacity and economic productivity, limiting women’s ability to provide income, food, care, and other resources for households. The offspring of short mothers tend to grow up stunted themselves and subsequently produce stunted children, creating a difficult-to-break, intergenerational cycle of stunting and reduced human capital [64,68]. The observation that HFI was associated with low maternal height underscores the importance of public health programs focusing on nutrition during critical times of growth: the first thousand days of life and the adolescent growth spurt period [64,69,70].

We also observed that the average weight and MUAC of women from food-insecure households were substantially lower than those from food-secure households. These findings align with the recent studies examining the association of food insecurity with the nutritional status of pregnant, lactating, or non-pregnant women in low-income settings [15,21,22,23]. Although our data did not show a statistically significant association of HFI with BMI, there was a trend for a negative relationship. Since both the average weight and height were lower among food-insecure women, the difference in BMI, which is, in fact, a ratio of the two, might have lost statistical significance. Moreover, several studies indicate that MUAC may be more sensitive to socioeconomic changes, better at detecting undernutrition, and superior prognostic indicator than BMI [71,72,73,74,75,76,77], and thus show a larger effect size. This can explain the statistical significance for MUAC and a lack of it for BMI in this study. However, future prospective cohort studies are needed to confirm the direction and strength of the dose-dependent relationship between HFI and the nutritional status of women. Women with low weight or MUAC in the first trimester are more likely to experience low GWG in the second and third trimesters [38,40], and subsequently are more likely to have preterm births and deliver LBW and small for gestational age (SGA) infants [38,39,40]. Furthermore, infants born to mothers with low GWG have a higher risk of neonatal morbidities and death [38,40]. 

Our results showed higher odds of all three negative mental states examined in this study, namely depression, anxiety, and stress, among the early-gestation women from food-insecure households compared to their food-secure counterparts. The inverse association identified in this study between HFI and mental health in pregnant women is consistent with existing literature [29,30,31,33,34]. Depression, anxiety, and stress are interrelated states, and they have a complex relationship with food insecurity. In pregnant women, HFI may provoke feelings of shame and desperation [78,79], leading to decreased social engagement [80,81], and triggering anxiety, distress, risk-taking behavior, impulsivity, aggression, dysfunctional relationships, and depression [82], as was discussed by Abrahams et al. [30]. Our findings strongly suggest that food insecurity can affect mental health in pregnant women. This is an important observation because sound mental health in pregnancy is crucial for optimal maternal and fetal health [83]. 

Pregnant women at risk of food insecurity should be identified at the earliest opportunity (preferably before conception) and prioritized for nutritional support in the form of food baskets or food vouchers through social safety net programs [84]. Women from low-income families should receive proper attention and support, especially during periods of external environmental shocks, such as the COVID-19 pandemic, as these conditions often exacerbate food insecurity to the extent that women cannot cope with it. In addition to the women, support should be extended to the family members, especially the children. In rural households of South Asia, including Bangladesh, women have limited involvement in making decisions and governing resources [85]. They are expected to be subservient to the husband or in-laws. Rural women rarely make informed choices about their daily activities, food purchases, dietary practices, self-care, and seeking healthcare for themselves and their children [85,86,87]. The feeling of powerlessness and inability to make an impact in the family place women at risk for poor mental health [88]. Promoting women empowerment may alleviate women’s mental health, food insecurity, and nutrition at the household level. 

Although this study used robust estimation techniques, its findings should be interpreted within the context of several limitations. Among the women from Matlab, who underwent screening in early pregnancy amid the COVID-19 pandemic, 10.6% were subjected to HFI. The prevalence is lower than the national prevalence (25%) of food-insecurity [3]. Although Matlab is generally considered to be less food-insecure than many parts of the country [89], it is important to realize that our study might have underestimated the prevalence of HFI because the screening dataset represents only the households from which pregnancies were reported. Due to limited access to electricity and mobile phones, many pregnancies from poor (and probably food-insecure) households were reported late during the pandemic and hence were not approached for screening and systematically excluded from the dataset. Moreover, amid the pandemic, pregnancy intendedness and number of incident pregnancies might have been selectively low in households experiencing food insecurity [90]. Finally, due to coronaphobia [91], some women refused to participate in screening during the pandemic. 

The cross-sectional nature of the data limited our ability to determine the temporality of the associations identified between HFI and women’s health. Some studies suggest that the relationship of food insecurity with nutritional status and mental health can be bidirectional [92,93,94]. However, investigating the causality or reverse-causality is beyond the scope of the present analysis. Another issue to consider is that nutritional status indicators, especially height and BMI, are related to long-term food security status. However, our study assessed households’ food security status over the preceding 30 days, which may not necessarily represent their long-term status. The study lacked adequate power to examine any dose-dependent effect of HFI due to the small sample sizes per categories of food insecurity and especially the rarity of severe food insecurity. However, the effect of food insecurity usually varies by severity and social policies and programs should also be customized according to the degree of food insecurity in a community [95]. This study investigated the association of women’s health with HFI; however, individual’s experience of food insecurity within the household may vary depending on age, sex, reproductive status, and other characteristics. Information on individual food consumption and dietary diversity could have helped, but the study lacked these data. Also, food security status may be misreported due to recall bias, respondent’s social status and desirability, and inherent limitations of food security measurement tools [44,96].

Although we included a number of key covariates in the propensity score model and later in the multivariable regression models, the dataset lacked any variable indicating family structure, which is a known driver of the complex linkage between food insecurity and women’s health [97]. However, there was no variability in marital status (all pregnant women were married), and all households were monogamous.

## 5. Conclusions

This study demonstrated that pregnant women from food-insecure households in rural Matlab were on average 2 cm shorter, 2 kg lighter and had 0.6 cm lower MUAC than their food-secure counterparts. HFI was associated with increased odds of depression, anxiety, and stress among these early-gestation pregnant women. Public health measures should focus on ensuring proper nutrition during the critical growth periods of life, pregnancy, and external environmental shocks, such as the COVID-19 pandemic, to mitigate the adverse effects of HFI on women’s health.

## Figures and Tables

**Table 1:** Sample characteristics before and after propensity score matching, by household food security status ^1^.

Characteristic	Food-Insecure	Food-Secure	*p*
Before matching (*n* = 672)			
Pregnant women, n	71	601	**-**
Age (years), mean (SD)	26.6 (6.4)	25.2 (5.8)	0.056
Religion, Muslim	90.1	83.7	0.158
Education (years), mean (SD)	6.6 (3.0)	8.4 (2.9)	<0.001
Parity			
Nulliparous	21.1	35.8	0.014
1 previous birth	45.1	38.9	0.318
≥2 previous births	33.8	25.3	0.123
Gestational age (weeks), mean (SD)	9.4 (1.6)	9.9 (1.8)	0.063
Chronic disease	5.6	4.8	0.766
Area			
Block A	32.4	27.6	0.398
Block B	39.4	29.8	0.096
Block C	14.1	22.6	0.099
Block D	14.1	20.0	0.236
Period			
Jan–Mar 2020	26.8	21.6	0.326
Apr–Jun 2020	19.7	16.6	0.514
Jul–Sep 2020	29.6	24.0	0.299
Oct 2020–Jan 2021	23.9	37.8	0.022
After matching (*n* = 665)			
Pregnant women, n	70	595	**-**
Age (years), mean (SD)	26.5 (6.5)	26.2 (5.8)	0.746
Religion, Muslim	90.0	89.7	0.959
Education (years), mean (SD)	6.7 (3.0)	6.8 (3.2)	0.837
Parity			
Nulliparous	21.4	22.6	0.874
1 previous birth	45.7	45.9	0.980
≥2 previous births	32.9	31.5	0.867
Gestational age (weeks), mean (SD)	9.4 (1.6)	9.5 (1.5)	0.934
Chronic disease	5.7	5.1	0.871
Area			
Block A	32.9	31.9	0.906
Block B	38.6	39.7	0.889
Block C	14.3	14.4	0.982
Block D	14.3	13.9	0.953
Period			
Jan–Mar 2020	27.1	24.3	0.707
Apr–Jun 2020	20.0	21.6	0.819
Jul–Sep 2020	28.6	31.0	0.751
Oct 2020–Jan 2021	24.3	23.0	0.863

^1^ All values indicate percentage unless otherwise indicated. Before matching, unweighted mean (SD) and proportion (percentage) are reported. After matching, mean (SD) and proportion (percentage) are weighted using the weight generated from the propensity score matching process. Before matching, *p* values are from *t*-tests based on an unweighted regression of each variable on the food security status. After matching, the regression is weighted using the weight generated from the propensity score matching process. The sample weight is not shown.

**Table 2:** Association of household food insecurity with nutritional status among early-gestation pregnant women in Matlab.

Outcome	Unadjusted		Adjusted ^1^	
	β (95% CI)	*p*	β (95% CI)	*p*
Height (cm)	−2.0 (−2.8, −1.3)	<0.001	−2.0 (−2.7, −1.2)	<0.001
Weight (kg)	−2.0 (−3.4, −0.6)	0.005	−2.0 (−3.4, −0.7)	0.003
BMI (kg/m^2^)	−0.3 (−0.8, 0.3)	0.365	−0.3 (−0.8, 0.2)	0.287
MUAC (cm)	−0.6 (−1.1, −0.1)	0.027	−0.6 (−1.1, −0.1)	0.010

^1^ Adjusted for age, religion, education, parity, gestational age, chronic disease, area (block), and period. Abbreviations: CI, confidence interval; BMI, body mass index; and MUAC, mid-upper arm circumference.

**Table 3:** Association of household food insecurity with mental health among early-gestation pregnant women in Matlab.

Outcome	Unadjusted		Adjusted ^1^	
	OR (95% CI)	*p*	OR (95% CI)	*p*
Depression	3.2 (1.8, 5.6)	<0.001	3.3 (1.8, 5.9)	<0.001
Anxiety	5.6 (3.5, 8.9)	<0.001	6.1 (3.7, 10.0)	<0.001
Stress	3.6 (1.4, 9.4)	0.009	4.8 (1.6, 14.2)	0.005

^1^ Adjusted for age, religion, education, parity, gestational age, chronic disease, area (block), and period. Abbreviations: OR, odds ratio; and CI, confidence interval.

## Data Availability

All aggregated data are provided within the paper. To protect the identification of participants derived from the composite of key study variables, some restrictions do apply to the primary data. These data can be made available from the Ethics Committees (RRC and ERC) of icddr,b for researchers who meet the criteria for access to confidential data. Please contact the Head of Research Administration at icddr,b (Armana Ahmed; aahmed@icddrb.org) for data policies and queries.

## Supplementary Materials

The following are available online at https://www.mdpi.com/article/10.3390/nu13124303/s1, Table S1: Logit model to generate propensity scores, Figure S1: Similarity of propensity score distributions before and after matching, Figure S2: Common support check, Figure S3: Standardized bias test for individual covariates, before and after matching.
