# Supplementary material for: Association of Household Food Insecurity with Nutritional Status and Mental Health of Pregnant Women in Rural Bangladesh
Source: Nutrients. 2021 Nov 28;13(12):4303. doi: 10.3390/nu13124303 (PMC8708397; doi:10.3390/nu13124303)
Supplement: Supplementary file 1 [file nutrients-13-04303-s001.zip › Table S1.pdf]

Table S1. Logit model to generate propensity scores.

Explanatory variable	Coefficient (95% CI)	P value
Age (years)	0.003 (-0.1, 0.1)	0.929
Religion		
Muslim	0.6 (-0.4, 1.5)	0.232
Hindu	Ref	
Education (years)	-0.2 (-0.3, -0.1)	<0.001
Parity		
Nulliparous	Ref	
1 previous birth	0.4 (-0.3, 1.1)	0.289
≥ previous births	0.2 (-0.8, 1.2)	0.719
Gestational age (weeks)	-0.1 (-0.3, 0.04)	0.136
Chronic disease		
Yes	0.1 (-1.0, 1.2)	0.890
No	Ref	
Block		
A	0.6 (-0.3, 1.4)	0.198
B	0.8 (-0.02, 1.7)	0.056
C	Ref	
D	0.1 (-0.9, 1.1)	0.863
Period		
Jan-Mar 20	0.5 (-0.2, 1.2)	0.178
Apr-Jun 20	0.8 (0.02, 1.6)	0.044
Jul-Sep 20	0.6 (-0.1, 1.3)	0.082
Oct 20-Jan 21	Ref	
Constant	-1.0 (-3.5, 1.4)	0.402
