# Supplementary figures and images for: Association of Household Food Insecurity with Nutritional Status and Mental Health of Pregnant Women in Rural Bangladesh
Source: Nutrients. 2021 Nov 28;13(12):4303. doi: 10.3390/nu13124303 (PMC8708397; doi:10.3390/nu13124303)

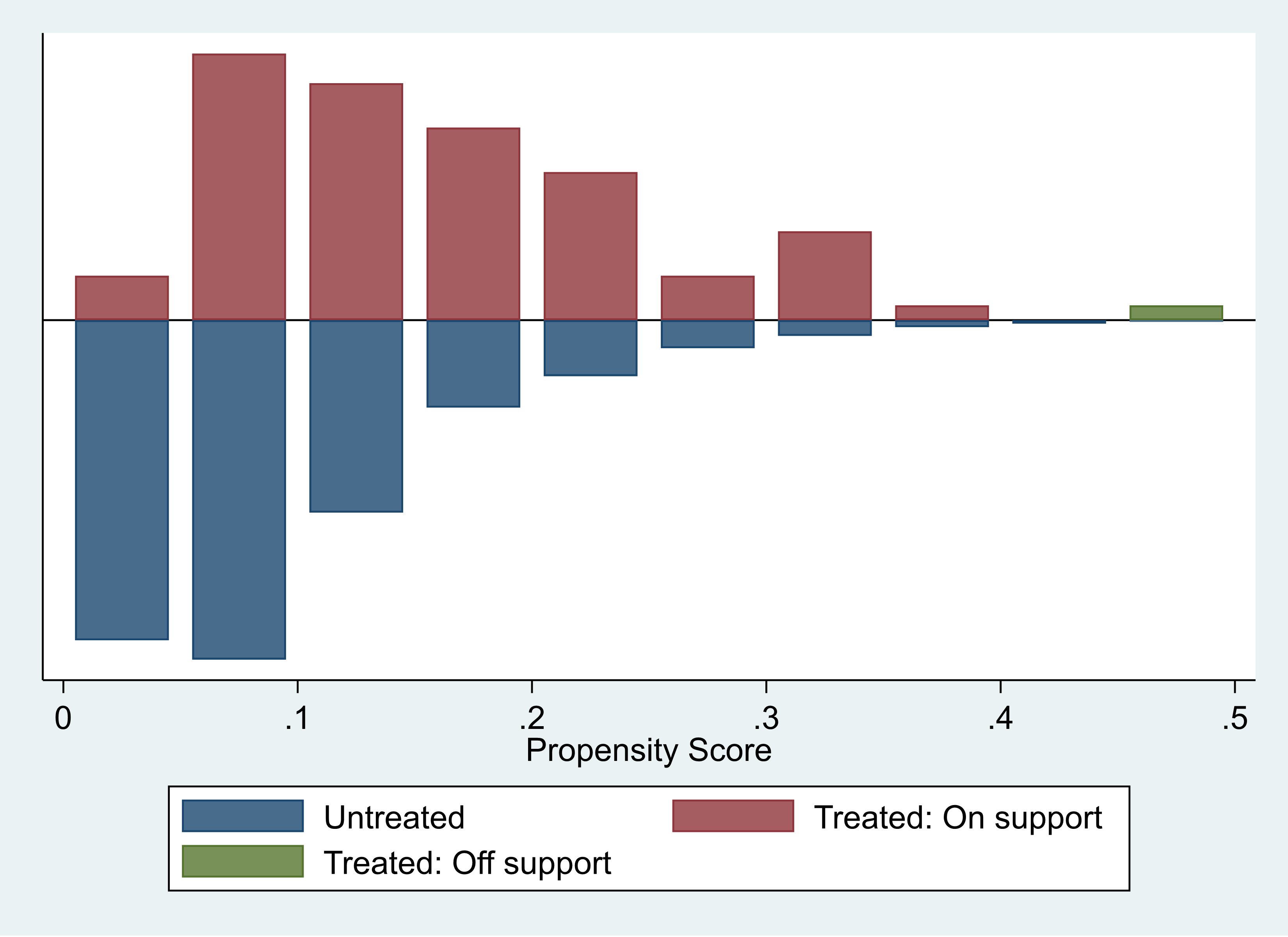

Supplement: Supplementary file 1 [file nutrients-13-04303-s001.zip › Figure S2.png]

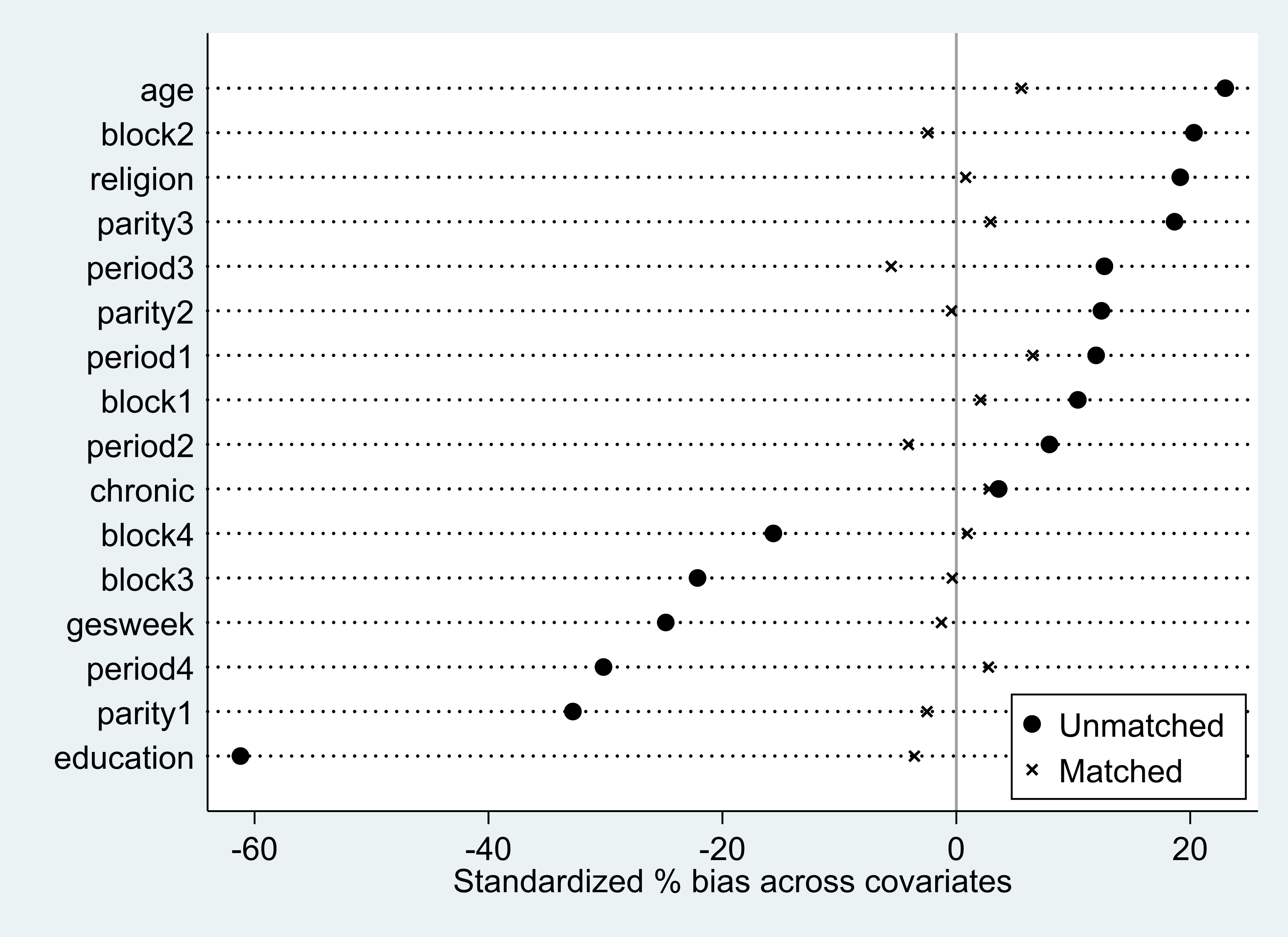

Supplement: Supplementary file 1 [file nutrients-13-04303-s001.zip › Figure S3.png]

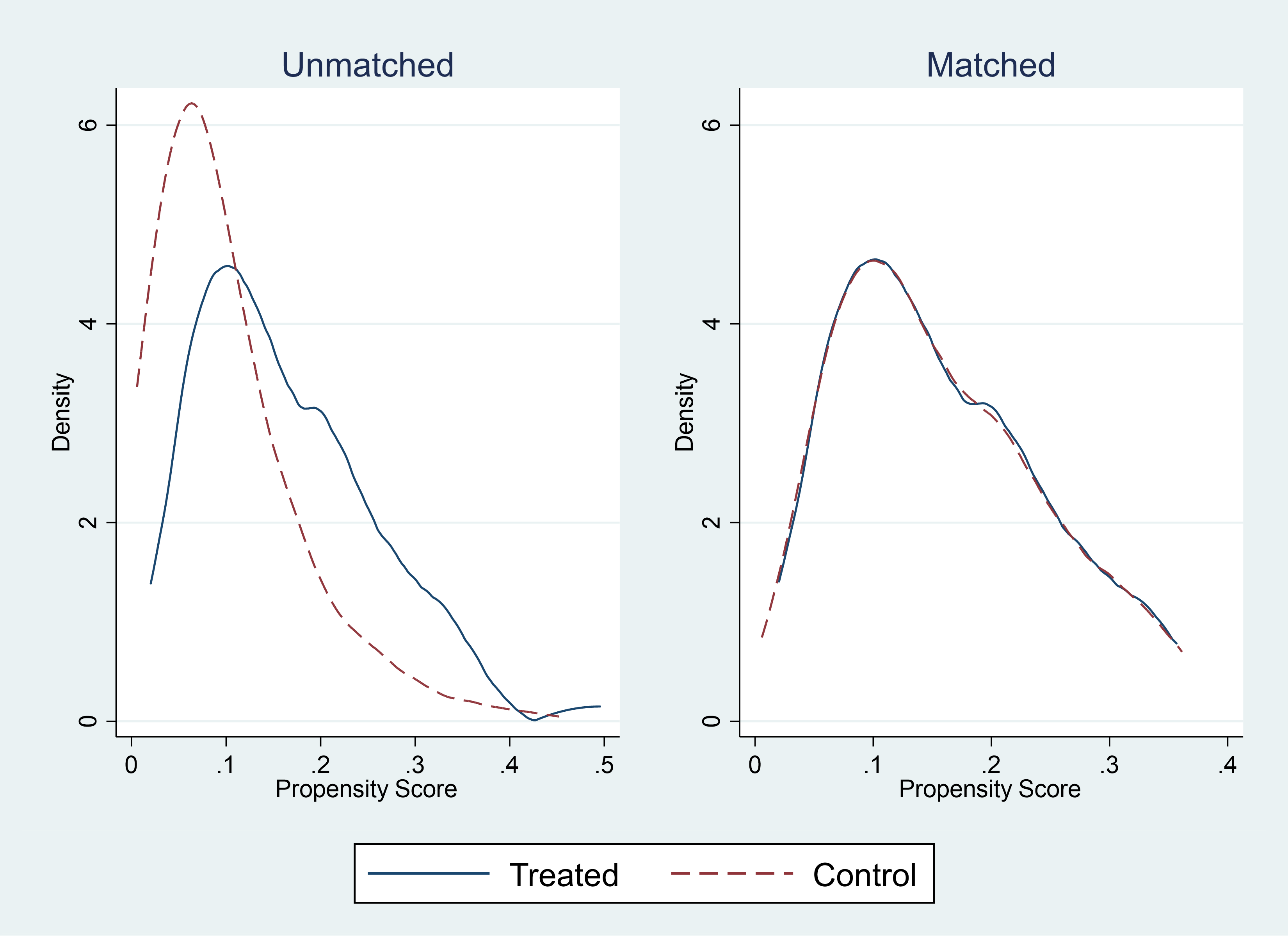

Supplement: Supplementary file 1 [file nutrients-13-04303-s001.zip › Figure S1.png]
